# Host Biologic Response to Acellular Dermal Matrices of Human and Porcine Origin

**DOI:** 10.1093/asjof/ojaf174

**Published:** 2026-01-07

**Authors:** Allen Gabriel, Rafael Gottenger, Nimesh Kabaria, Li-Ting Huang, Patrick Leamy, Victoria Stefanelli, Wenqi Zuo, Eric Stec, Maryellen Gardocki-Sandor, Hui Xu

## Abstract

**Background:**

Acellular dermal matrices (ADMs) originate from various tissues and are manufactured by different processes, which can influence material properties that affect host response upon implantation.

**Objectives:**

The aim of the study was to compare material properties and host responses to ADMs derived from human or porcine dermis.

**Methods:**

The morphology of commercially available ADMs (human-derived AlloDerm [hADM], porcine-derived Strattice Pliable [pADM-S], and porcine-derived Artia [pADM-A]) was evaluated through bright-field and scanning electron microscopy and compared with unprocessed human and porcine dermis. Collagen melting temperatures were assessed through differential scanning calorimetry. Host responses to ADMs were assessed in rat (pADM-A and pADM-S) and nonhuman primate (NHP) models (pADM-A, pADM-S, and hADM). Histologic responses were evaluated for inflammation, fibroblast infiltration, and revascularization.

**Results:**

Morphologically, the extracellular matrix structures of hADM, pADM-A, and native human dermis were similarly loose, whereas the structures of pADM-S and native porcine dermis were tighter. Collagen melting temperatures were similar across all samples. Following 20 h exposure to collagenase enzyme, hADM retained the most undigested collagen, followed by pADM-A, then pADM-S. Following 2 and 4 weeks of implantation in the rodent and NHP models, pADM-A yielded the most favorable host response with ample cell infiltration, vascularization, and minimal inflammation vs other implanted ADMs. Minimal-to-moderate inflammatory responses were observed for all materials; hADM and pADM-A demonstrated the lowest responses.

**Conclusions:**

In both short-term preclinical implantation models, comparable morphologies and biochemistry of pADM-A and hADM allowed for similarly favorable host responses vs pADM-S. All ADMs assessed demonstrated responses consistent with a favorable regenerative mechanism of action.

**Level of Evidence: 4 (Therapeutic):**

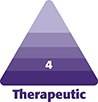

Use of acellular dermal matrices (ADMs) in surgical procedures has become widespread since they were first introduced in the 1990s.^1-3^ ADMs are used throughout the body for plastic and reconstructive procedures to provide soft tissue closure and coverage across large and small defects, including for improved aesthetic outcomes, support in hernia repair, and aiding wound management.^4,5^

ADMs originate from various native tissue sources and are manufactured using different processes before use in surgical procedures.^6^ Commercially available ADMs are typically derived from human, porcine, or bovine dermis.^4^ After the native tissue is harvested, it undergoes processing to remove the cells, which leaves behind a decellularized scaffold.^4,5^ Various processes can be used to achieve decellularization, including physical, chemical, and biochemical methods.^7^

The source of the ADM and the decellularization process may impact the immunologic-based inflammatory response following implantation. Ineffective or incomplete removal of cells and cellular components can result in an antigenic response leading to inflammation.^7^ The structural and biochemical integrity of the tissue may also be affected by decellularization processes, which may aid or prevent the host tissue from integrating into the ADM following implantation.^7^

The objective of this study was to compare the host responses in different animal models to ADMs derived from dermal tissues of human or porcine origin and to determine the potential effect of species, antigenicity, and structural and biochemical properties on host response.

## METHODS

### Study Design

The ADMs (all Allergan Aesthetics, an AbbVie Company, Branchburg, NJ) assessed in these studies included 1 human-derived ADM (hADM [AlloDerm]) and 2 porcine-derived ADMs (pADM-S [Strattice Pliable] and pADM-A [Artia]). These studies were conducted in compliance with the principles of the Declaration of Helsinki, and human-derived materials evaluated were previously consented for use in scientific research.

### In Vitro Assessment

The morphology of ADMs was assessed histologically using hematoxylin and eosin (H&E) and picrosirius red (PSR) staining and immunohistochemically through staining for Collagen types I and VI. Scanning electron microscopy (SEM) was also used to compare collagen fibril structure and configuration among the ADMs as well as to native human and porcine dermis. Differential scanning calorimetry (DSC) was used to assess collagen onset melting temperature (*T*_0_) as well as denaturation and modifications to the tissue matrix. Susceptibility of the ADMs to digestion by collagenase was also assessed.

### In Vivo Assessment

ADM samples were created from a single piece of ADM from a single lot and implanted subcutaneously into Lewis rats (1 × 1 cm) or into nonhuman primates (NHPs) (1.5 × 1.5 cm) to evaluate host response. Only porcine-derived materials, pADM-S and pADM-A (*n* = 2 each), were implanted into Lewis rats to minimize confounding xenogeneic factors. Samples from all 3 ADMs were randomly distributed and implanted into the NHP model, with *n* = 8 samples implanted per animal. ADM samples were explanted and evaluated at 2 and 4 weeks postimplantation from both the rodent and NHP models using blunt dissection, and care was taken to retain any associated host tissue with the explanted samples for analysis. Explanted ADM samples from the NHP model were stained histologically with H&E to evaluate overall host response and immunohistochemically for vimentin, CD31 antigen, and CD68 antigen for specific detection of fibroblasts, vascular endothelial cells, and macrophages, respectively.

### Acellular Dermal Matrices and Host Response Analysis

Following explantation from both animal models, ADMs and surrounding tissues were fixed in 10% neutral buffered formalin for at least 24 h, processed for routine histology, and stained with H&E. Morphology of the ADMs was assessed, and a semiquantitative comprehensive histologic host response score was assigned by a subject matter expert who was blinded to the identity of each explant. A cumulative histologic response score was assigned to each sample that took into account inflammation, fibroblast cell infiltration, and revascularization of the ADM. This scoring system was developed based on previous observations of ADM samples explanted from preclinical implantation models and reflects the observed inverse relationship between inflammatory cell infiltration into the ADM as opposed to fibroblast-like cell infiltration, coupled with host revascularization of the ADM (Table 1).^8-10^ Immunohistochemically stained samples were evaluated qualitatively to corroborate identification of specific cell populations observed through H&E. For the primate study, systemic antibody induction was measured through enzyme-linked immunosorbent assay (ELISA) conducted on serum samples drawn weekly following ADM implantation.

**Table 1.** Histologic Response Score Definitions. Cumulative Scoring System Based on Histologic Host Response Observations From Previous ADM Implantation Studies^8-10^

Score^a^	1	2^b^	3	4^b^	5	6^b^	7	8^b^	9
Observations	Significant inflammation		High inflammation		Moderate inflammation		Minimal inflammation		No inflammation
Observations	No repopulation^c^		Minimal repopulation^c^		Moderate repopulation^c^		High repopulation^c^		Superior repopulation^c^
Observations	No vasculature^d^		Minimal vasculature^d^		Moderate vasculature^d^		High vasculature^d^		Superior vasculature^d^

ADM, acellular dermal matrix.

^a^Higher scores represent the most favorable response and most remodeled ADMs.

^b^Descriptions for 2, 4, 6, and 8 are not included because they are midpoints between the odd-numbered scores.

^c^Repopulation is characterized by quantity and distribution of fibroblast-like cells (ie, depth of penetration into the thickness of the cross-section).

^d^Vasculature is characterized by quantity and nature (ie, types of cells around the vessels; eg, if vessels are surrounded by inflammatory cells, this is less desirable).

## RESULTS

### In Vitro Results

**Figure 1.**
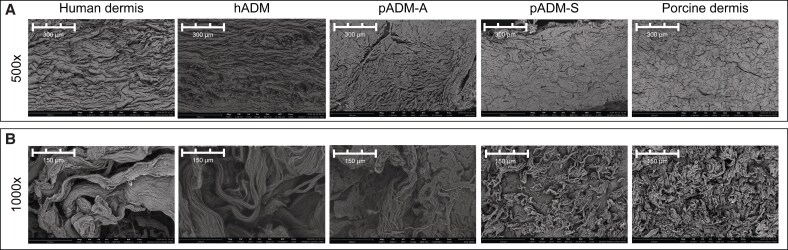
Scanning electron micrographs of extracellular matrix ultrastructure of native, unprocessed human dermis; hADM; pADM-A; pADM-S; and native, unprocessed, porcine dermis. The overall extracellular matrix architecture for the ADMs and native human and porcine dermis are shown in (A), and the collagen fibril ultrastructures are shown in (B). ADM, acellular dermal matrix; hADM, human-derived ADM (AlloDerm); pADM-A, porcine-derived ADM (Artia); pADM-S, porcine-derived ADM (Strattice Pliable).

Scanning electron micrographs of the extracellular matrix (ECM) ultrastructure of all 3 ADMs, as well as native, unprocessed human and porcine dermis, are shown in Figure 1. The morphology of native human dermis and hADM appeared similar, as did the architectures of native porcine dermis and pADM-S, with minimal structural changes evident because of decellularization and sterilization processes employed to manufacture the ADMs (Figure 1A). The ECM architecture of pADM-A, also derived from porcine dermis, however, demonstrated similar morphology to native human dermis and hADM (Figure 1A). Compared with both native human and porcine dermis, the overall integrity of the collagen fibrillar structure for hADM, pADM-S, and pADM-A was maintained through manufacturing processing (Figure 1B).

**Figure 2.**
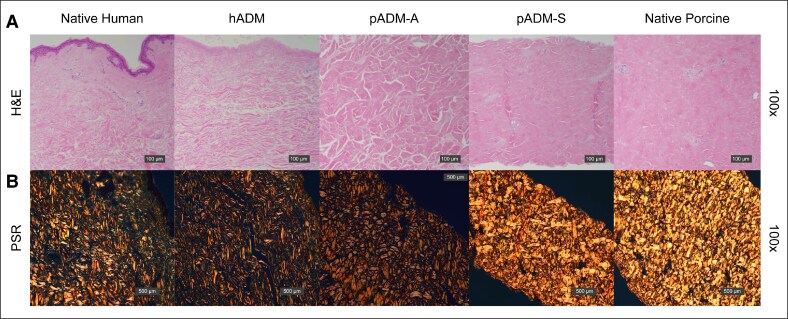
Histological assessment of native, unprocessed human dermis; hADM; pADM-A; pADM-S; and native, unprocessed porcine dermis. H&E staining of all 3 ADMs and native human and porcine dermis are shown in (A). PSR staining of all materials tested is shown in (B). ADM, acellular dermal matrix; H&E, hematoxylin and eosin; hADM, human-derived ADM (AlloDerm); pADM-A, porcine-derived ADM (Artia); pADM-S, porcine-derived ADM (Strattice Pliable); PSR, picrosirius red.

**Figure 3.**
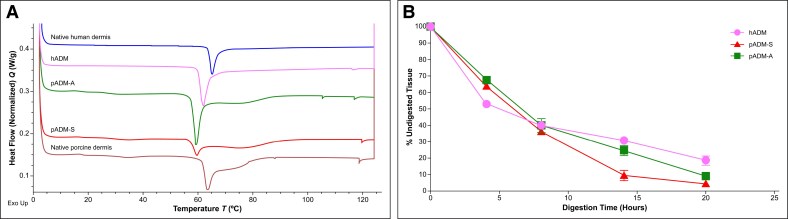
Differential scanning calorimetric (DSC) analysis (A) and collagenase assay (B). DSC heat flow thermograms for native, unprocessed human dermis; hADM; native, unprocessed porcine dermis; pADM-A; and pADM-S are shown in (A). Collagenase digestion of all 3 ADMs is shown in (B). ADM, acellular dermal matrix; hADM, human-derived ADM (AlloDerm); pADM-A, porcine-derived ADM (Artia); pADM-S, porcine-derived ADM (Strattice Pliable).

Histological assessment corroborated SEM findings that hADM and pADM-S retained a similar structure to native human and native porcine dermis, respectively (Figure 2). However, there was a difference in apparent fiber compactness observed between pADM-A and native porcine dermis. In particular, pADM-A collagen fibers appeared to be less densely packed and more closely resembled human dermis than porcine dermis (Figure 2). Collagen types I and VI were detected immunohistochemically in all samples tested. Collagen onset melting temperature measurements, as determined by DSC, were similar across all samples, with native human and native porcine dermis having the highest melting temperatures of 63.8°C (standard deviation = 0.1°C) and 61.3°C (0.3°C), respectively, and hADM, pADM-A, and pADM-S having somewhat lower onset melting temperatures (59.9°C [0.2°C], 57.3°C [0.1°C], and 57.2°C [0.2°C], respectively; Figure 3A).

The 3 ADMs demonstrated susceptibility to collagenase over time; all ADMs rapidly degraded similarly during the first 8 h. Although only pADM-S maintained this rapid rate of degradation throughout the study, both hADM and pADM-A continued to degrade at a more gradual rate over the next 12 h until all 3 ADMs were reduced to 20% or less of the original mass (Figure 3B). At assay conclusion (20 h), the final amount of remaining undigested tissue was greater for hADM compared with pADM-A and pADM-S (18.6% ± 2.5%, 9.3% ± 0.3%, and 4.6% ± 0.5%, respectively).

### Rodent Host Response

**Figure 4.**
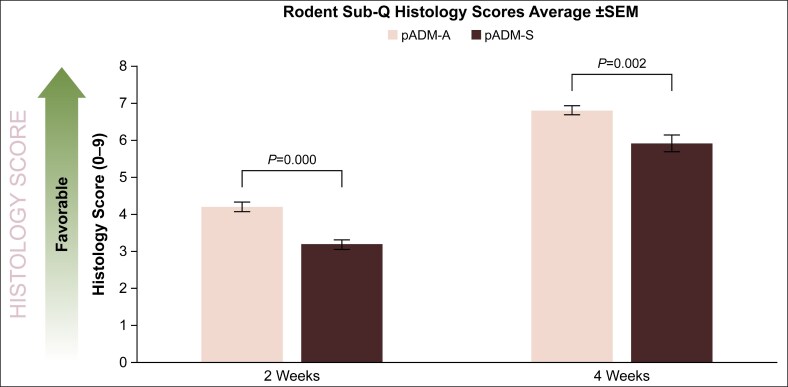
Histological assessment of pADM-A and pADM-S after 2 and 4 weeks of implantation in the rodent model. Scores are a cumulative summation of fibroblast infiltration, vascularization, and inflammatory cell presence as detailed in Table 1. *P* values based on the Kruskal–Wallis test were calculated for each time point (significance level, *P* < .05). ADM, acellular dermal matrix; pADM-A, porcine-derived ADM (Artia); pADM-S, porcine-derived ADM (Strattice Pliable); SEM, standard error of the mean; Sub-Q, subcutaneous.

Comparison of host responses to pADM-A and pADM-S implanted subcutaneously in a rodent model demonstrated that overall localized histologic response scores for pADM-A at 2 and 4 weeks postimplantation were 4.2 ± 0.1 and 6.8 ± 0.1, respectively, which was significantly more favorable than the host responses at 2 and 4 weeks postimplantation for pADM-S (3.2 ± 0.1 [*P* = .000] and 5.9 ± 0.2 [*P* = .002], respectively; Figure 4). Notably, both types of porcine-derived ADMs implanted in the rodent model demonstrated significantly increased, therefore improved, host response scores between 2 and 4 weeks of implant duration, demonstrating minimal-to-moderate inflammation, as well as moderate-to-high levels of both fibroblast infiltration and vasculature by the conclusion of this short-term study.

### Nonhuman Primate Host Response

**Figure 5.**
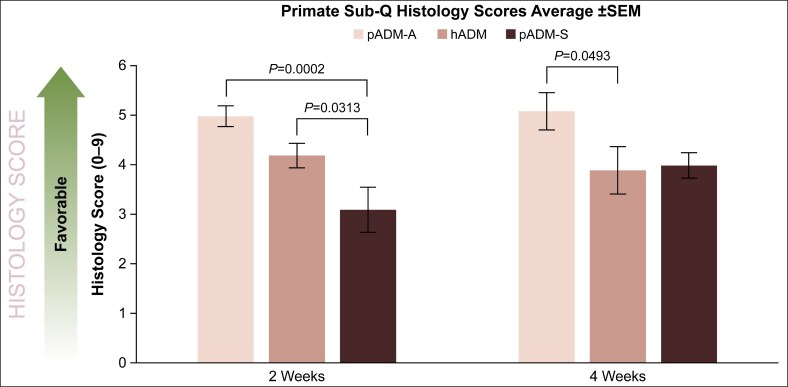
Histological scoring assessment of pADM-A, hADM, and pADM-S after 2 and 4 weeks of implantation in the nonhuman primate model. Scores are a cumulative summation of fibroblast infiltration, vascularization, and inflammatory cell presence as detailed in Table 1. ADM, acellular dermal matrix; hADM, human-derived ADM (AlloDerm); pADM-A, porcine-derived ADM (Artia); pADM-S, porcine-derived ADM (Strattice Pliable); SEM, standard error of the mean; Sub-Q, subcutaneous. *P* values based on the Kruskal–Wallis test were calculated for each time point (significance level, *P* < .05).

**Figure 6.**
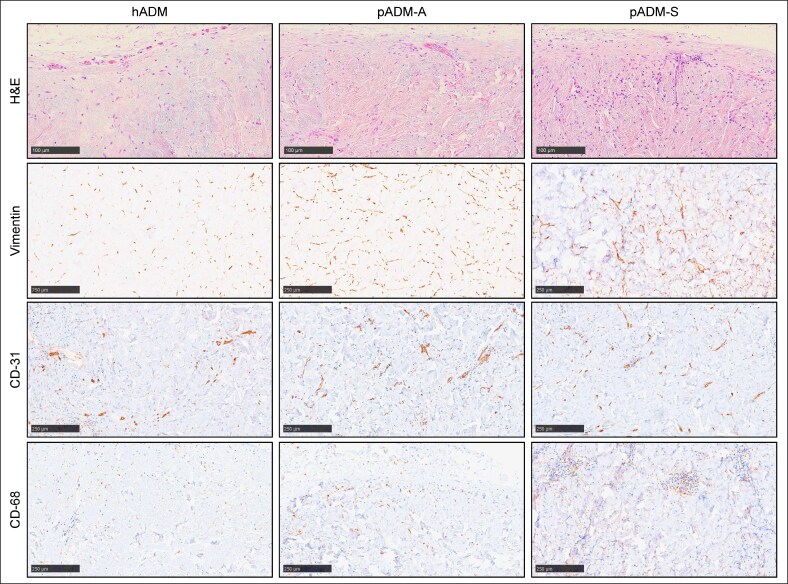
Histologic host response to pADM-A, pADM-S, and hADM after 4 weeks of implantation in the nonhuman primate subcutaneous implant model. 200× magnification. H&E staining for cumulative semiquantitative scoring of overall host response, as shown in Figure 5. Immunohistochemical staining for vimentin, CD31, and CD68 for qualitative assessment of the presence of fibroblasts, vascular endothelial cells, and macrophages, respectively. ADM, acellular dermal matrix; H&E, hematoxylin and eosin; hADM, human-derived ADM (AlloDerm); pADM-A, porcine-derived ADM (Artia); pADM-S, porcine-derived ADM (Strattice Pliable).

In the NHP model, localized histologic response scores were most favorable for pADM-A at 2 and 4 weeks following implantation (5.0 ± 0.2 [*P* = .0002 vs hADM] and 5.1 ± 0.4 [*P* = .0493 vs hADM], respectively), followed by hADM (4.2 ± 0.3 and 3.9 ± 0.5, respectively), and then pADM-S (3.1 ± 0.5 [*P* = .0313 vs hADM] and 4.0 ± 0.3, respectively; Figure 5). Immunohistochemical staining corroborated the presence of fibroblasts, vascular endothelial cells, and macrophages in explanted samples of all 3 ADMs (Figure 6). In addition, both hADM and pADM-A elicited minimal immune responses systemically as demonstrated through ELISA serum antibody analysis (1- to 2-fold and 1- to 4-fold titer increase, respectively, at 4 weeks), whereas pADM-S elicited a transient minimal-to-moderate systemic response at 4 weeks (4- to 32-fold titer increase).

## DISCUSSION

In vitro assessments in this study analyzed the structural, thermal, and biochemical properties of hADM, pADM-A, and pADM-S to evaluate the impact that decellularization and sterilization may have had on the integrity of the ADMs when compared with native, unprocessed human and porcine dermal tissue. The effects of species of origin, antigenicity, and material properties, including structural and biochemical properties, of the 3 different ADMs on host response in rodent and NHP subcutaneous implantation models were also investigated.

In vitro assessments in this study demonstrated that hADM and pADM-S ECM ultrastructures were similar to their native corresponding tissue of origin, human and porcine dermis, respectively. Among these samples, human tissues had a more open fibrillar architecture relative to porcine tissues, which is consistent with previous findings demonstrating comparative SEM and histological analysis of human and porcine tissues.^11,12^ Additionally, small-angle X-ray scattering quantitatively demonstrated increased isotropic orientation of collagen fibrils in human tissues compared with the strongly bimodal orientation of collagen fibrils in porcine tissues.^12^ Interestingly, in this study, pADM-A demonstrated an ECM ultrastructure that more closely resembled human dermis, despite it being derived from porcine dermis. The dissimilarity in ECM ultrastructure between pADM-A and porcine dermis and the similarity of pADM-A to native human dermis, as demonstrated by SEM, H&E, and PSR staining showing compactness and birefringence of the collagenous matrix, indicated a modification to the overall collagen fiber arrangement during the decellularization processing of native porcine dermis to pADM-A, which did not occur with pADM-S processing or with processing of native human dermis to hADM.

The ability to process native porcine dermis to have a similarly loose architecture as the human dermis, as seen with pADM-A in this study, without sacrificing collagen fibril integrity is important because tissue architecture and compactness can have an impact on cellular infiltration. A study that used identical processing methods for human and porcine dermis demonstrated that more fibroblasts infiltrated the more open architecture of the human ADM over 4 weeks compared with the more closed architecture of the porcine dermis.^11^ Additionally, although fibroblasts infiltrated both ADMs, the human ADM had a more open structure after 4 weeks compared with baseline, whereas the porcine ADM maintained its tightly packed architecture.^11^ These results indicate that the initial openness of an ADM may influence the ability of both cells and vasculature to infiltrate and cause matrix remodeling. Therefore, there may also be an accelerated biological interaction of cells with pADM-A because of its altered and more open structure compared with pADM-S.

In vitro assessments of collagen content, thermal stability, and susceptibility to digestion by collagenase enzyme demonstrated retained collagen integrity in all 3 processed ADMs. Immunohistochemical analysis for Collagen types I and VI indicated retained presence of each in all ADMs tested. Additionally, although the ECM ultrastructure of pADM-A more closely resembled human dermis than porcine dermis morphologically by SEM and histologic methods, collagen melting temperatures of the ADMs were more similar to their species of origin. Specifically, the human-derived tissues (native human dermis and hADM) tended to have higher onset melting temperatures than their corresponding nonprocessed (native porcine dermis) or processed (pADM-S and pADM-A) porcine-derived counterparts. The consistency of thermal properties within species indicates a lack of denaturation of the collagen protein integrity because of processing, as corroborated by high-magnification SEM (1000×).

An in vitro collagenase assay was conducted to understand the differences in rates of ADM digestion. Although there were differences in architecture and thermal properties, all ADM samples initially exhibited similar rates of digestion. However, there appeared to be an inverse correlation between the level of ADM architectural openness and the rate of degradation. In particular, hADM had a somewhat slower digestion rate than either pADM-A or pADM-S, and pADM-S had the greatest amount of digested collagen, followed by pADM-A. Overall, the hADM sample retained the greatest amount of undigested tissue following enzyme exposure. These results indicate that within the controlled setting of a benchtop collagenase assay where all samples were exposed to identical enzyme concentrations per mass of tissue, thermal properties like *T*_0_ may have had greater influence on the rate of digestion than ADM fibril architecture. In the in vivo setting, however, cells are the primary source of digestive enzymes, and thus, differing rates of cellular infiltration may affect digestion rates in combination with tissue thermal properties. The differing influence of the in vitro vs in vivo setting indicates the necessity of using preclinical models.

In the rodent model, both pADM-A and pADM-S elicited minimal-to-moderate inflammatory response, moderate-to-high fibroblast cell infiltration, and moderate-to-high blood vessel formation between 2 and 4 weeks, demonstrating a regenerative mechanism of action that increased throughout the duration of the study. Although there was not a statistically significant difference, there was a trend of pADM-A having a more favorable histological score than pADM-S. In the NHP study, despite being porcine derived and a xenogeneic tissue implant, pADM-A yielded moderate inflammation, fibroblast cell infiltration, and vascularization at the 2- and 4-week time points. The host responses to pADM-A were somewhat higher but similar to hADM. For pADM-S, a significantly lower overall histology score was observed at 2 weeks compared with the other ADMs, but it demonstrated a significant increase in score by 4 weeks, indicating a lowered inflammatory response, improved fibroblast cell infiltration, and vascularization comparable to hADM. Specific immunohistochemical staining for fibroblasts with vimentin, blood vessel formation with CD31, and macrophages with CD68 corroborated these findings.

The targeted biochemical processing regimen used to manufacture pADM-A, in addition to modifying the overall tightness of the porcine dermal fibers without ECM denaturation, may contribute to the lower early inflammatory response of pADM-A compared with the somewhat higher, although transient, early inflammatory response observed for pADM-S. This is in contrast to previous studies demonstrating that other commercially available porcine-derived processed matrices elicited more elevated, persistent systemic immune responses than the ADMs assessed in this study when placed in the same NHP model.^8^ Whereas all ADMs assessed in this study demonstrated satisfactory incorporation, inflammatory, and vascularization responses in these preclinical models, the results indicate that pADM-A had a more open architecture compared with pADM-S that may have allowed for an enhanced rate of cell infiltration that likely contributed to the more favorable host responses to pADM-A.

Despite the frequent use of ADMs in plastic and reconstructive surgery, there is a gap in the literature for direct comparison of patient outcomes from and immune responses to different ADMs in the clinical setting.^13^ A systematic review of ADM use in ventral hernia repair that included 23 studies assessing 15 different ADMs, including pADM-S and hADM, concluded that there were no significant differences in rates of recurrence or surgical site infection among biologic ADMs, which demonstrates that the tissue source of an ADM may not directly impact these clinical outcomes.^14^ However, there is evidence that different tissue processing methods used in the manufacturing of ADMs impact the ECM structure, which may lead to different immunologic responses and clinical outcomes. Two cohort studies and a database study assessing outcomes in ventral hernia repair and abdominal wall repair with biologic ADMs, including pADM-S and hADM, demonstrated fewer complications with pADM-S and hADM compared with other porcine-, bovine-, and human-derived ADMs.^15-17^ More clinical studies assessing immunologic and host responses to ADMs derived from different sources and/or created with different processing techniques are needed.

Limitations include a lack of longer-term preclinical implantation as well as clinical testing in humans for immune response to the ADMs in the study. Further investigation of immune responses to pADM-A in clinical trials is needed to confirm if results reported here will also be observed in plastic and reconstructive procedures in humans.

## CONCLUSIONS

All 3 ADMs assessed in this study demonstrated favorable histology and host response that may be suitable for use in surgical procedures depending on surgeon preference. When all 3 ADMs were compared in both in vitro studies and in vivo rodent and NHP subcutaneous implant models, pADM-A demonstrated a decrease in porcine dermis ECM tightness, without corresponding denaturation, and improved early host response.

These results indicate that pADM-A, with more similar material properties to human dermis, can result in an enhanced early biologic response. Additionally, although the host response was somewhat improved for pADM-A at these early time points, all 3 ADMs were shown to support a regenerative mechanism of action.
